# Tonal modulation influences on musical sight-reading

**DOI:** 10.3389/fpsyg.2026.1736951

**Published:** 2026-03-04

**Authors:** Yumo Zhang, Olivia Podolak Lewandowska, Spencer Jones, Mark A. Schmuckler

**Affiliations:** Department of Psychology, University of Toronto Scarborough, Toronto, ON, Canada

**Keywords:** motor control, music cognition and perception, music performance, perception-action approach, sight-reading

## Abstract

Musical sight-reading requires decoding visual notation into coordinated motor actions, making it an invaluable paradigm for studying the perceptual and motor representations underlying perception–action coupling. Two experiments examined the impact of tonal modulations on sight-reading, having pianists perform melodies varying in the tonal distance of their modulation (no modulation, close modulation, mid modulation, and far modulation). Experiment 1 presented melodies in a random order, whereas Experiment 2 presenting melodies blocked by condition (no modulation melodies first versus modulating melodies first). In both studies, analyses of performance errors revealed increased errors from the initial key to the subsequent key. Additionally, both experiments found gradated tonal distance effects, with far modulations producing the largest difference in error rate between initial and subsequent keys, no modulations producing the least difference, and close and mid modulations falling in between the two. Finally, both experiments observed a spike in error rates, with errors peaking at the transition point from the initial to the subsequent key. Of note is that Experiment 1 showed this (albeit non-significant) pattern for the no modulation melodies, suggesting that pianists developed expectations for key modulation irrespective of its occurrence. Experiment 2 confirmed this hypothesis, demonstrating no change in error rates for no modulation melodies when pianists performed these melodies prior to experiencing the modulating melodies. Together, these results support a perception-action account of piano performance, and suggest intriguing new directions for research on real-time music performance.

## 1 Introduction

Music performance is a complex behavior that engages numerous psychological processes in its practitioners (Palmer, 1997, 2005, 2013). These processes cut across a wide array of abilities, including multisensory integration involved in visual, auditory, and haptic perception, motor behavior and control, interpersonal interaction and synchrony, emotional production and perception, and even anxiety regulation. As evidence of the plethora of psychological components underlying music performance, Parncutt and McPherson’s (2002) edited volume on this topic contains no less that nine chapters on the “subskills of music performance,” with these components ranging from perceptual processing of visual and auditory input (McPherson and Gabrielsson, 2002) to motor learning and practice (Barry and Hallam, 2002) to memory processes (Aiello and Williamon, 2002) to structural (Friberg and Battel, 2002) and emotional communication (Juslin and Persson, 2002) to body movement (Davidson and Correia, 2002). Accordingly, musical performance represents a microcosm of general psychological processing. It is unsurprising, therefore, that investigations of musical performance have been one of the fastest growing (Gabrielsson, 2003; Tirovolas and Levitin, 2011) and most highly cited (Cohen and Schmuckler, 2023) areas in music psychology.

One framework adopted for understanding music performance involves a focus on the explicit perception-action relations required for this behavior (Maes et al., 2013; Novembre and Keller, 2014; Pfordresher, 2006, 2019; Schaefer, 2014). According to this approach, performance of notated music is foundationally structured by the recoding of visual symbols necessary for the structure and planning of physical movement, as well as the auditory perception of sounds produced by these actions. These perceptual and motor components recursively interact, forming a continuous perception-action loop in which perceptual input influences motor behavior, with this motor behavior then influencing the subsequent planning and execution of this movement, which then creates new perceptual input, and so on.

Within the realm of auditory information, probably the most well-known, and thoroughly studied example of this approach involves work on delayed auditory feedback, investigated by Kulpa and Pfordresher (2013), Pfordresher (2003, 2005, 2008, 2019), Pfordresher and Dalla Bella (2011), and Pfordresher and Palmer (2002, 2006) as well as others (Bradshaw et al., 1971; Finney, 1997; Finney and Palmer, 2003; Finney and Warren, 2002; Gates and Bradshaw, 1974; Gates et al., 1974; Havlicek, 1968). Such research has demonstrated that the motor control involved in producing musical passages is affected by the availability and timing of auditory information arising from such performances. As some examples of this influence, Pfordresher and Palmer (2002) found that delayed auditory feedback affected the timing variability of performances of isochronous melodies, with the extent of timing variations influenced by whether the delay produced binary or non-binary subdivisions of the underlying beat structure. Similarly, Pfordresher and Dalla Bella (2011) found that even simple tapping of an isochronous rhythm was affected by delayed auditory feedback, with these effects evident in both timing variability as well as the velocity of finger movements. Overall, this research represents a paradigmatic example of a perception-action approach in understanding the complexities inherent in music performance.

Although not as thoroughly studied, within the visual domain one arena for investigating perception-action relations involves sight-reading (Banton, 1995; Kopiez and Lee, 2008; Lehmann and Ericsson, 1993, 1996; Lehmann and Kopiez, 2016; Lehmann and McArthur, 2002; Lewandowska and Schmuckler, 2019; Mishra, 2014a, 2014b; Perra et al., 2021; Schön and Besson, 2002; Sloboda, 1974; Wolf, 1976). Because musical sight-reading involves performing passages from a score with no or little prior practice or experience with the notation, this behavior provides an ideal context for exploring the use of visual information in perception-action loops, as sight-reading requires performers to immediately decode visual input, and integrate both current and upcoming information with corresponding motor behavior. Research on sight-reading has focused on three different issues: eye movements during sight-reading, structural factors influencing sight-reading, and factors related to sight-reading skill, including how to teach and improve sight-reading (see Lewandowska and Schmuckler, 2019, for a discussion). Of these areas, explorations of the structural factors influencing sight-reading music (see reviews by Kopiez and Lee, 2008; Mishra, 2014a) are especially illuminating for understanding perception-action relations, given that such factors, by definition, involve critical visual and auditory input to be translated, in some form, into motor activity.

Research on the impact of structural components on musical sight-reading has itself explored a wide range of factors, including visual and auditory feedback (Delogu et al., 2019; Finney and Warren, 2002; Pfordresher, 2005, 2006), pitch, rhythmic, and handedness complexity (Fine et al., 2006; Gregory, 1972; Gudmundsdottir, 2010; Lewandowska and Schmuckler, 2019), and phrase structure (Ahken et al., 2012; Arthur et al., 2016; Madell and Hébert, 2008; Mainenti et al., 2011; Sloboda, 1977). One particularly important structural component that has been explored involves the tonal structure of the to-be-performed passage. Briefly described, tonality refers to the organization of the 12 chromatic tones around a central reference pitch (Krumhansl, 1990, 2000; Krumhansl and Cuddy, 2010; Schmuckler, 2004, 2009, 2016, 2023). In Western music, these chromatic tones form a hierarchy, with the central reference pitch, called the tonic, positioned at the top of the hierarchy, and the remaining tones occupying differing positions in this hierarchy depending upon their theoretic relatedness to the tonic. Tonalities can be built on any of the 12 chromatic notes, and are generally one of two types – major or minor – that vary in the specifics of their internal hierarchical relations. A wealth of research in the field attests not only to the psychological reality of these “tonal hierarchies” (Krumhansl, 1990, 2000; Krumhansl and Kessler, 1982; Krumhansl and Shepard, 1979), but the impact of tonality on a wide array of psychological behaviors, including online processing of musical information, memory for musical materials, and the performance of musical passages (see Lewandowska and Schmuckler, 2019; Schmuckler, 2016, 2023, for reviews).

Tonality has similarly featured prominently in sight-reading research (Alexander and Henry, 2012; Fine et al., 2006; Lewandowska and Schmuckler, 2019; MacKenzie et al., 1986). For instance, MacKenzie et al. (1986) had performers sight-read tonal and atonal (music that is not tonally structured) passages, and found increased rhythmic variability in the performance of a target passage in atonal, relative to tonal passages. Similarly, Fine et al. (2006) had performers sight-read Bach chorales manipulated such that the melody of the passages were either tonal or atonal, with either a tonal or atonal harmonic accompaniment, and found that introducing atonality into the passages increased errors in performance. Most recently, Lewandowska and Schmuckler (2019) had pianists sight-read passages varying in their tonality (major tonality, minor tonality, atonality) and texture (simultaneous versus successive tone onsets, unimanual versus bimanual performance), and found that both factors significantly influenced sight-reading accuracy. Together, these findings highlight that tonal structure plays an important role in the perception-action organization of music performance.

Despite demonstrating the importance of tonality as a structural principal in piano performance and sight-reading, this work is limited in certain respects. For instance, these findings do not necessarily indicate the continuous interactive nature of perceptual and action systems during this behavior. Specifically, although tonal information can be abstracted from the to-be-performed musical scores, because this tonal structure stays constant across this context, motor performance might only be constrained by information gathered at the start of the performance, and not as a result of continuous perceptual monitoring and updating. Put more simply, a static tonal structure across the length of a passage does not actually require continuous perception-action feedback throughout performance to demonstrate influences of this structural factor, although clearly continuous monitoring is required for the actual production of the to-be-performed notes.

One way to address this limitation would be to explore sight-reading within contexts that require explicit continuous monitoring of the tonal structure through the entire passage. Such a situation could be achieved by examining sight-reading in passages that change in their tonal organization. In fact, shifting tonal organizations, called “tonal modulations” or “key changes,” are extremely common in Western music, with there being no limit to the number of modulations that can occur in a piece, nor any constraint with respect to the distance, in tonal space, over which such movements can occur (Korsakova-Kreyn and Dowling, 2014; Krumhansl and Kessler, 1982). According to some authors, tonal modulations are essential to the aesthetic properties of a piece of music (Brattico et al., 2013; Brattico and Pearce, 2013; Brattico et al., 2017; Korsakova-Kreyn and Dowling, 2014).

Over the years, researchers have examined the psychological impact of tonal modulations, primarily focusing on listeners’ percepts of key movement (Cook, 1987; Cuddy and Thompson, 1992; Farbood, 2016; Marvin and Brinkman, 1999; Spyra et al., 2021; Spyra and Woolhouse, 2023; Thompson and Cuddy, 1989, 1992, 1997; Woolhouse et al., 2016). This work has explored multiple issues, including the ability to track tonal modulations throughout a piece of music (Chew, 2002, 2007, 2014; Krumhansl, 1990; Schmuckler, 2023; Schmuckler and Tomovski, 2005), factors affecting the perception of tonal closure (Marvin and Brinkman, 1999; Spyra et al., 2021; Spyra and Woolhouse, 2023), the timescale on which previously heard tonal information disappears in memory (Cook, 1987; Farbood, 2016; Janata et al., 2003; Janata et al., 2002; Woolhouse et al., 2016), the effect of tonal modulation on subjective time (Firmino and Bueno, 2008; Firmino et al., 2009), the role of tonal distance and the direction of tonal movement on tonal percepts (Cuddy and Thompson, 1992; Lewandowska, 2019; Lewandowska and Schmuckler, in preparation; Thompson and Cuddy, 1989, 1992, 1997), and neural correlates of tonal modulations (Janata, 2007; Janata et al., 2003; Janata et al., 2002; Koelsch and Friederici, 2003; Koelsch et al., 2003). As an oversimplified summary, this work suggests that listeners can accurately track modulations throughout a piece of music, that the tonal distance of modulations can influence percepts of modulations in some circumstances, that tonal modulations are encoded in systematic patterns of cortical activation, and that there seems to be little expectation for, or even recognition of, whether a piece returns to the initial key in which it started.

Of note is the fact that virtually no work has examined the impact of tonal modulation on performances of musical pieces. Although Thompson and Cuddy (1997) did employ a performance manipulation in their research, this study focused on whether performance expression enhanced listeners’ perceptions of tonal movement. As such, the impact of key movement on the musical performances remains an open question.

Taken together, this review highlights an obvious avenue for exploring the flexibility of perception-action feedback in music performance. Specifically, examining the impact of key movement during a musical sight-reading task provides a window into the operation of perception-action integration presumably underlying music performance. Towards this end, a pair of experiments investigated the impact of modulation on music performance, specifically exploring the effect on pianists’ sight-reading of a modulation from an initial tonality in the beginning of a passage to a subsequent tonality at the end of a passage. Assuming that the sight-reading of a musical excerpt requires some form of initial perceptual and motor representations, the occurrence of a modulation would similarly necessitate the modification of both schemas. The necessity of modifying both perceptual and motor schemas could produce multiple consequences on performance, resulting in a set of predictions for performance. For instance, one outcome of changing representations might be that, at the transition point between the two keys, because performers must adapt to the changing perceptual input by altering their motor performance, there could be an observable disruption in their performance. Put more simply, performance should noticeably deteriorate during the key transitions.

Second, and related to this first prediction, because performers must adjust their underlying representations, there could be an observable impact on performance that continues for a period of time into the new key section. Although one would anticipate that this impact would eventually dissipate over time, such an effect could well produce a disruption of performance in the subsequent key section, relative to the initial key section.

Finally, a third consequence of the requirement of shifting perceptual and motor representations due to tonal modulation can be seen in considering the impact on performance of modulations varying in their distance in tonal space between initial and subsequent keys. Building on work highlighting the importance of tonal distance in perceptions of modulations (Cuddy and Thompson, 1992; Lewandowska, 2019; Lewandowska and Schmuckler, in preparation; Thompson and Cuddy, 1989, 1992, 1997), if sight-reading of modulating passages does require modifying perceptual and motor representations, then modulations varying in their tonal distance should similarly, and differentially, affect performance. Specifically, more distant modulations, which require more complex realignment in perceptual and motor schemas, should lead to more errors in sight-reading, relative to less distance modulations.

## 2 Experiment 1: sight-reading of modulating melodies: tonal distance effects

Experiment 1 provided an initial test of the question of the impact of tonal modulation on performers’ sight-reading of short musical passage. To examine this question, this study draws from research examining listeners’ percepts of tonal modulations (Cuddy and Thompson, 1992; Farbood, 2016; Lewandowska, 2019; Lewandowska and Schmuckler, in preparation; Thompson and Cuddy, 1989, 1992, 1997; Woolhouse et al., 2016), within the methodological context of recent work on tonality and sight-reading (Lewandowska and Schmuckler, 2019).

**Figure 1:**
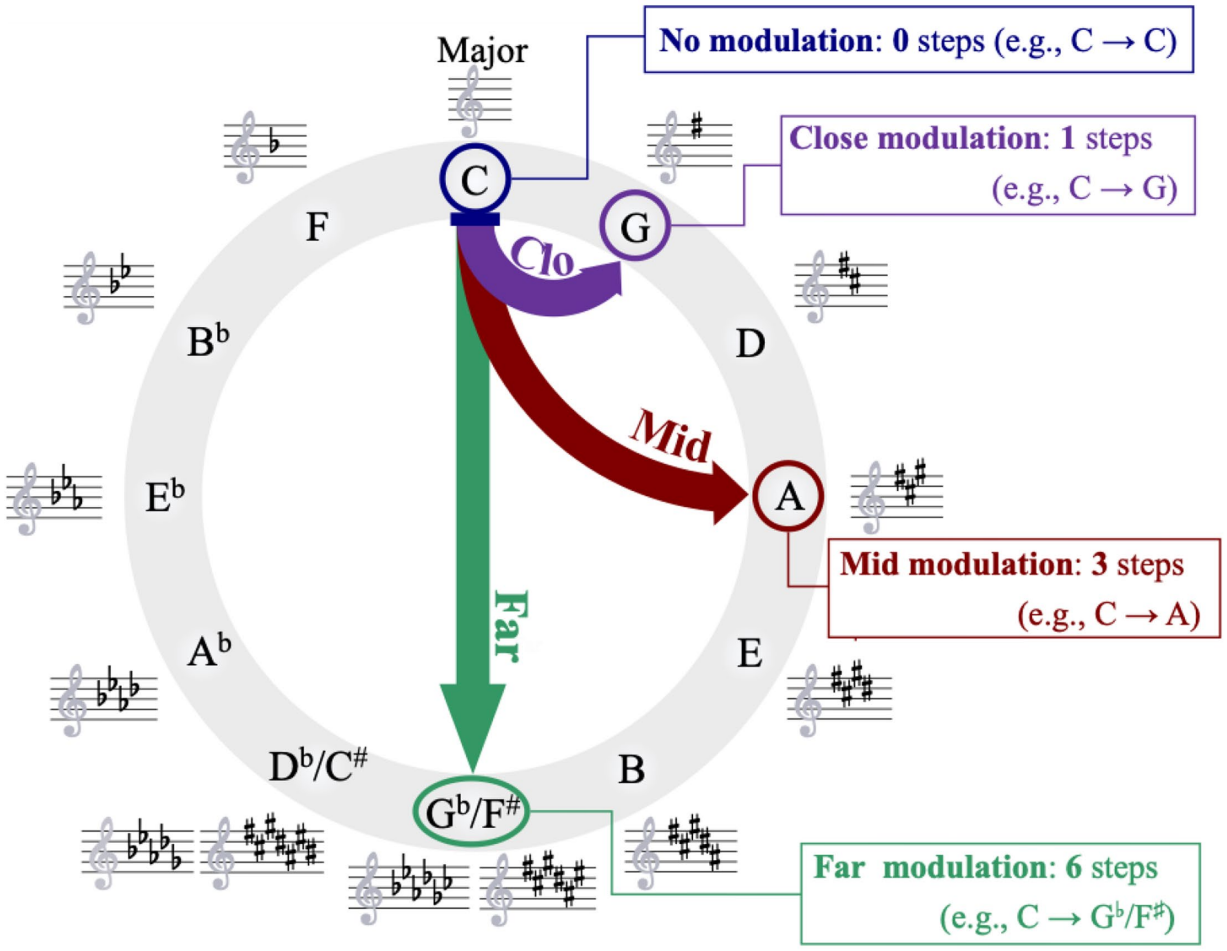
The circle of fifths, showing distance in tonal space for major keys. Examples of enharmonic spellings for two keys are provided. The different tonal modulation conditions (close, mid, far) are shown as arrows around this circle.

One critical issue underlying exploration of this question involves delineating how best to characterize the degree of tonal distance present in modulating stimuli. Within a Western music-theoretic framework, one means of characterizing the degree of modulation involves mapping the distance between the initial and subsequent tonalities on the “circle of fifths.” This arrangement, shown in Figure 1, portrays the relations between the 12 chromatic pitches and the major and minor keys built on them, positioned in a circular sequence in which neighboring tonalities are separated by the musical interval of a perfect fifth (i.e., seven semitones). One advantage to this arrangement is that it spatially portrays the musical relation between the tonalities, with the number of steps between different tonalities indicative of the similarity or relatedness between the tonalities. Thus, adjacent tonalities along this circle (tonalities one step apart, such as C and G, Db and Ab, and so on) are highly related tonalities, whereas maximally distance tonalities along this circle (tonalities six steps apart, such as C and F#/Gb or A and Eb) are highly unrelated tonalities. Indeed, a wealth of research (see Bartlett and Dowling, 1980; Krumhansl and Kessler, 1982; Shepard, 1982a, 1982b, for classic references) attests to the perceived reality of these key relations. Accordingly, this arrangement provides a convenient quantification of the degree of tonal distance for modulating passages.

### 2.1 Method

#### 2.1.1 Participants

Twenty-five trained pianists (19 females; mean age = 20.64 years, *SD* = 2.50 years) were recruited from the University of Toronto Scarborough student population. Participants received either course credit in a psychology course or a $30 Amazon Gift card for participating. Eligibility requirements for this study included having a Grade 7 sight-reading ability based on guidelines established by the Royal Conservatory of Music (RCM), or an equivalent musical institution. At this grade level, RCM sight reading assessments require individuals to sight-read unfamiliar musical materials either as lead sheets (with chord symbols), or simple two handed passages of music of 8–12 measures in length, with these passages varying in their tonality (in major or minor keys of up to three flats or sharps), time signatures (simple and compound meters), and rhythmic structure (The Royal Conservatory, 2022). Grade 7 was selected as a conservative inclusion threshold to ensure fluent performance of the experimental stimuli (whose tonal, metric and rhythmic complexity fell below the upper limits of the Grade 7 sight-reading demands) while avoiding unnecessarily restrictive eligibility criteria that would limit participant recruitment. Additionally, by Grade 7, pianists would have completed technical requirements (e.g., scales, arpeggios, etc.) encompassing all major and minor scales, ensuring familiarity with the full range of tonal materials used in this study. Participants reported an average of 14.64 (*SD* = 3.03) years of playing, 10.68 (*SD* = 3.08) years of formal instruction, 5.52 (*SD* = 6.06) hours of weekly practice, with a modal level of 10 for their highest certificate in piano performance.

#### 2.1.2 Materials

**Figure 2:**
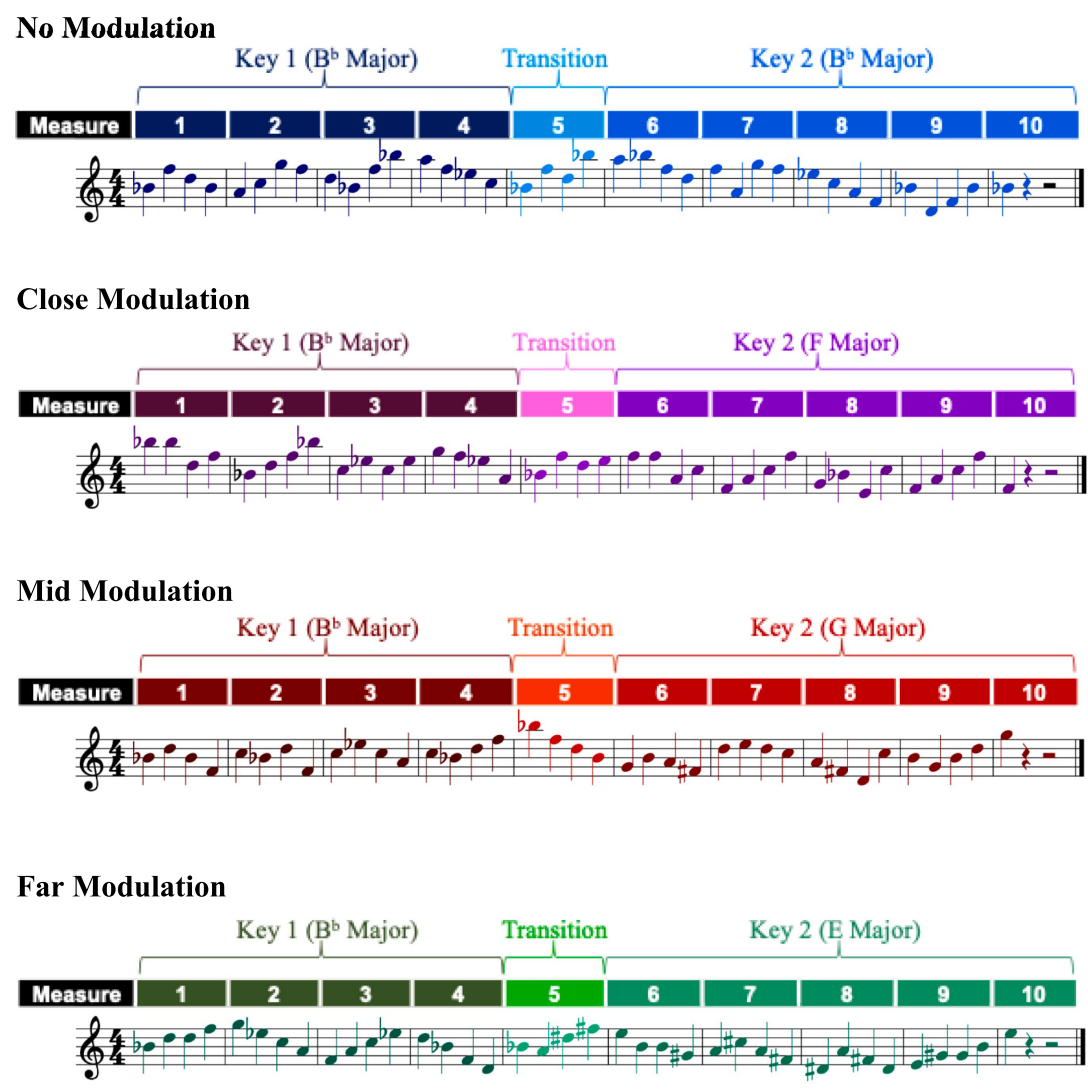
Sample melodies in the no modulation, close modulation, mid modulation, and far modulation conditions in Experiment 1.

The stimuli for this study consisted of 10-measure melodies in 4/4 time, written for the right hand, composed entirely of quarter notes, with the final measure containing only a single note. All melodies were structured such that the first four measures (measures 1–4) instantiated an initial tonality (called “Key 1”), followed by a transition measure (measure 5) in which the melody modulated to a new key (called “Transition”), and ended with five measures (measures 6–10) that instantiated this new tonality (called “Key 2”). Sample melodies for this experiment are shown in Figure 2, denoting the Key 1, Transition, and Key 2 sections, as a function of the principal experimental manipulation (see below). All melodies were presented devoid of key signatures, with all accidentals (sharps and flats) written directly into the score.

The principal manipulation of this study involved the distance, in tonal space, of the modulation in the melody. This manipulation consisted of four conditions: “no modulation,” “close modulation,” “mid modulation,” and “far modulation.” Figure 1 displays these four conditions schematically, with respect to the circle of fifths. No modulation melodies did not contain any movement in tonal space; Key 1 was the same as Key 2. Close modulation conditions involved a movement of one clockwise step along the circle of fifths. As already discussed, tonalities differing by a single step are as closely related as possible in Western music (Aldwell and Schachter, 2002; Rosen, 1972; Walton, 2010). Mid modulation conditions involved a movement of three clockwise steps along the circle of fifths. Tonalities separated by this distance represent less common modulations in Western music (Aldwell and Schachter, 2002; Laitz, 1996; Walton, 2010). Finally, far modulation conditions involved a movement of six steps along the circle of fifths. In Western music, such modulations are very uncommon (Aldwell and Schachter, 2002; Walton, 2010).^1^

Each of the four conditions were comprised of 12 melodies to be sight-read by participants. Each of these melodies started in one of the 12 unique major tonalities based on the chromatic set, and ended in a unique major tonality. For example, in the no modulation condition, one stimulus began in C major and ended in C major (see Figure 1), another stimulus began and ended in C#/Db major, another began and ended in D major, and so on. Similarly, in the close modulation condition, one stimulus began in C major and ended in G major (see Figure 1), one stimulus began in C#/Db major and ended in G#/Ab major, and so on. And as a final example, in the far modulation condition, one stimulus began in C major and ended in F#/Gb major (see Figure 1), one began in C#/Db major and ended in G major, and so on. The 12 exemplars in each of the 4 modulation conditions thus produced 48 stimulus melodies in total.

The decision to employ one-handed melodic lines, devoid of key signatures, was driven by a few factors. First, had we included key signatures, this information could have not only provided inadvertent *a priori* cueing to performers of the tonal modulation condition of a given to-be-performed stimulus, but also of the location of the position of the transition between keys within the stimulus. Along these lines, the tonal modulation would have been discernible from the nature of the key signatures, with the no modulation melodies containing only a single key signature, far modulations containing two divergent key signatures, likely employing differing accidental types (e.g., sharps in one key signature and flats in the other), with close and mid modulation conditions falling between these two extremes. Moreover, the placement of the second key signature in the score (possibilities in this regard include the beginning of the transition measure, within the transition measure itself, or the beginning of the second key section) would have indicated where the key transition occurred. Although these aspects would be likely adduced by performers through their performances, and over the course of the experiment, it was believed important to not provide such information on an *a priori* basis, available through a cursory visual inspection of the scores.

As for employing one-handed melodic lines, this choice was driven by multiple factors. First, and pragmatically, based on work by Lewandowska and Schmuckler (2019), one-handed melodic lines seemed to offer an optimal level of performance challenge. As described earlier, in Lewandowska and Schmuckler performers sight read both one-handed (single melodic lines) and two-handed (homophonic and polyphonic) four measure musical passages, producing error rates between 6% (one-handed) and 19% (two-handed). Given that the current study intended to use passages over twice as long as this previous work, there was a concern that required two-handed performances would pose overwhelming sight-reading challenges for performers.

Second, and converging with the just expressed concern regarding the use of key signatures, the exact content of two-handed passages was also felt to be potentially problematic. Given the utility of using melodies as stimuli, in terms of being able to create a key transition section, as opposed to a single transition (e.g., a pivot) event, two-handed performance would have required the use of either two independent right- and left-hand melodic lines, or a right-hand melodic line with a left-hand harmonic accompaniment. The former is not ideal in that it would have required performers to sight read the equivalent of (simplified) two-part inventions, whereas the latter would have re-introduced idealized harmonic information that would be the virtual equivalent of including key signature information. Given all of these reasons, it was determined that employing one-handed melodies were the optimum choice for stimuli.

All stimuli were rendered as musical scores, written in treble clef. Different types of accidentals (i.e., sharps versus flats) were employed both across and importantly, within stimuli, depending upon the specific enharmonic spelling of the two keys sounded. As an example, for the far modulation stimulus Db – G, Key 1 (Db) employed flats, whereas Key 2 (G) employed sharps. The mixed use of both sharps and flats within a score is common in Western tonal music, and would not be seen as unusual by participants. Finally, all melodies were written within a two-octave span ranging from C_4_ to C_6_.

#### 2.1.3 Apparatus

Participants performed these melodies on a Yamaha S-80, full-sized weighted, digital keyboard, connected to a Windows based PC via a Digidesign Mbox 2 Pro interface. Both MIDI and audio data were recorded using Cakewalk SONAR Studio (v8.0). An electronic metronome set to 140 beats per minute (BPM) provided a tempo cue throughout all trials to encourage participants to maintain a constant tempo during performance, to play continuously through each trial, without stopping to correct errors, and to prevent performers from playing so slowly as to not make any errors. Auditory feedback from the keyboard was input into a Mackie 1604 mixing console and presented to performers through a pair of Project Studio 5 speakers placed to their left and right.

#### 2.1.4 Procedure

Prior to beginning the experiment, participants were simply told that this study was exploring musical sight-reading, provided written informed consent, and filled out an online questionnaire collecting relevant demographic and musical background information. Specifically, participants were told that they would be sight-reading a series of melodies with their right hand only, and that all of these melodies would be 10 measures long in 4/4 time, consisting only of quarter notes. Participants were also instructed that there would be no key signatures for these melodies, and that any accidentals that occurred should be treated as typical for musical scores (i.e., the occurrence of an accidental remained in effect for all subsequent occurrences of that specific note in the measure, but not beyond). Participants were told they would hear a metronome during the trial, and were asked to perform the passage with an emphasis on pitch accuracy (play the indicated note), while maintaining the tempo of the metronome. Thus, performers were asked to not “correct” errors in sight-reading, but to simply continue their performance regardless of the occurrence of errors; such a constraint is common in sight-reading contexts.

At the start of each trial, participants received 10 s to scan the to-be-performed melody. Once this time elapsed, performers heard two measures (i.e., eight beats) of a metronome count-in, after which they began their performance. After performing a given melody, performers were given a few seconds break, and then began the next trial. The 48 trials (4 modulation conditions × 12 exemplars) were randomized for all participants. Halfway through the experiment (i.e., after completing the 24th trial), participants were offered a break if they desired. After finishing the 48 trials, participants were debriefed as to the nature of the stimuli and the purpose of the study. The entire experimental session lasted 60–90 min in total.

### 2.2 Results

#### 2.2.1 Data preprocessing

**Figure 3:**
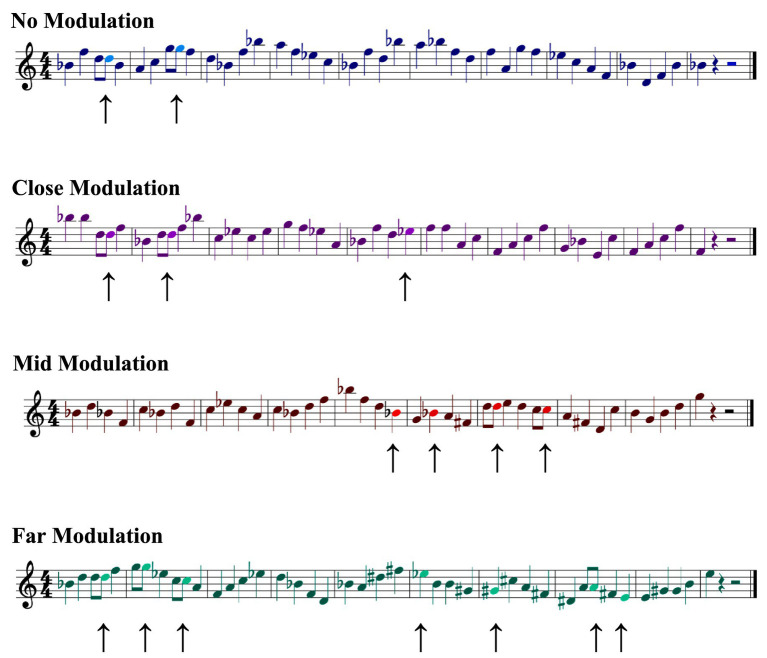
Sample performed melodies in the no modulation, close modulation, mid modulation, and far modulation conditions in Experiment 1. Performance errors are indicated with arrows (↑). Note that added notes have been written as eighth notes, rather than quartertones; this rhythmic deviation was employed to facilitate comparison with Figure 2.

All performances were output as musical scores (using the aforementioned SONAR software) in common Western music notation. Pitch errors for all trials were manually coded by comparing the pitch events of the performed melody with the original musical score. Pitch errors were based on performance error coding schemes adapted from Palmer and van de Sande (1993, 1995), and were defined as any played note that was different from what was notated in the score (a substitution or added note) or a note omitted altogether during sight-reading. Figure 3 shows examples of the performances for the sample melodies presented previously (Figure 2), with errors in performances indicated.

Pitch errors were aggregated for each performance for each participant for subsequent analysis. The initial step in this process involved summing the total number of errors occurring on a measure-by-measure basis; for this analysis, measure 10 was included with the errors for measure 9. The summed errors were converted to percent errors by dividing by the number of to-be-performed notes in each measure and multiplying by 100. The average error rates on a measure basis were then averaged across the 12 exemplars for each of the no modulation, close modulation, mid modulation, and far modulation conditions for each participant. Finally, aggregate error rates representing the different sections based on their sounded tonalities were calculated. For this measure, the average error rates for measures 1–4 (Key 1), corresponding to the first tonality, and for measures 6–9 (Key 2), corresponding to the second tonality, were created; error rates for measure 5 (Transition), the transition measure between the tonalities were kept separate. This process created three sections for each modulation conditions: Key 1, Transition, and Key 2. Note that for the no modulation condition, these sections all represent the same tonality. Thus, this division was based on the temporal parallels between these melodies and the remaining three conditions, not on the inherent tonality of these sections *per se*.^2^

#### 2.2.2 Data analysis

Error rates were analyzed using a two-way Analysis of Variance (ANOVA), with the within-subjects factors of *Tonal Section* (Key 1, Transition, Key 2) and *Modulation Condition* (no modulation, close modulation, mid modulation, far modulation). This analysis found normality was violated for all combinations of these two variables, based on the Shapiro–Wilk test. However, because ANOVAs are robust to such violations (Schmider et al., 2010) without inflating Type 1 error, we decided not to transform the data. Additionally, Machley’s test indicated that sphericity was violated for the two-way interaction between *Tonal Section* and *Modulation Condition* (*p* = 0.01). Accordingly, for this effect, the Greenhouse–Geisser correction was applied to the relevant degrees of freedom, and is reported here.

**Figure 4:**
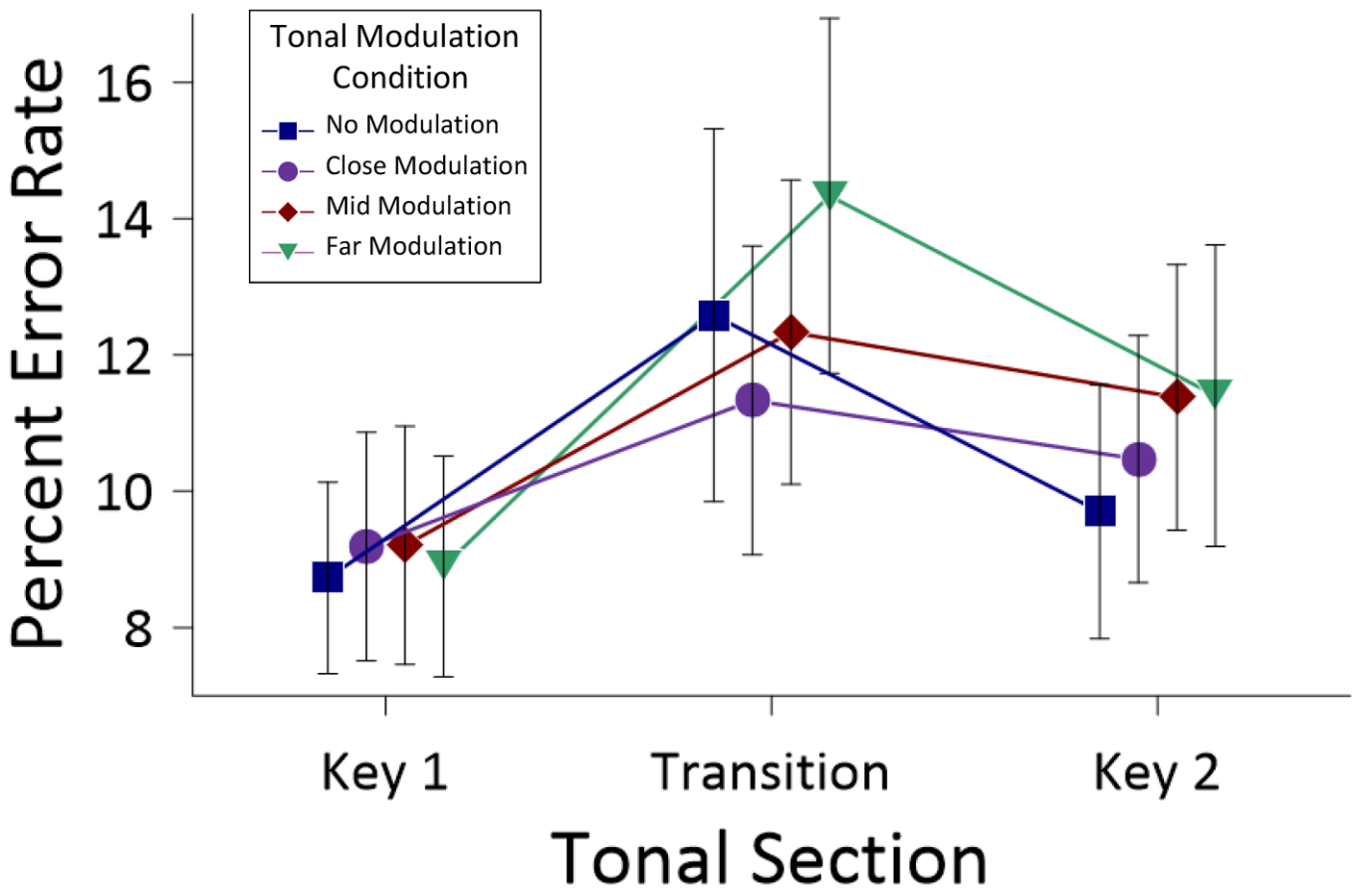
Percent error (and SEs) for the *Tonal modulation* conditions, as a function of *Tonal Section*, for Experiment 1. This figure is presented for information only; the two-way interaction was not significant.

Figure 4 presents the error rates (and SEs) as a function of *Tonal Section* and *Modulation Condition*. This ANOVA revealed a main effect of *Tonal Section*, *F*(2,48) = 10.53, *MSE* = 31.50, *p* < 0.001, *partial η^2^* = 0.30, but no main effect of *Modulation Condition*, *F*(3,72) = 1.56, *MSE* = 16.32, *n.s.*, nor any interaction between the two factors, *F*(4.29,102.94) = 1.32, *MSE* = 16.65, *n.s*. To examine differences in error rates as a function of the main effect of *Tonal Section* (i.e., collapsed across *Modulation Condition*), *post hoc* tests using Holm-Bonferroni corrections were conducted for all pairwise comparisons. The Holm–Bonferroni procedure is a multiple comparison correction that adjusts significance thresholds in a stepwise manner, controlling the family-wise error rate while providing greater statistical power than the standard Bonferroni correction. These comparisons revealed significant differences between error rates for all pairs of means: Key 1 (*M* = 9.00, *SE* = 1.54) versus Transition (*M* = 12.65, *SE* = 2.31), *t*(24) = −3.91, *p* = 0.002, Key 1 versus Key 2 (*M* = 10.34, *SE* = 1.94), *t*(24) = −2.74, *p* = 0.023, and Transition versus Key 2, *t*(24) = 2.42, *p* < 0.023.

Despite the non-significant interaction, a series of *post hoc* analyses with Holm-Bonferroni corrections examined the differences between these tonal sections for each modulation condition individually. The results of these comparisons appear in Table 1. Generally, error rates for Key 1 were less than for the Transition section for the no modulation (marginally significant), and the mid and far modulation conditions (significant). When comparing Key 1 and Key 2, error rates for Key 2 exceeded those for Key 1 for both mid and far modulations. Finally, comparisons of error rates between the Transition section and Key 2 were less systematic, with differences for the no modulation condition (again marginally significant) and the far modulation condition, but not for the close or mid modulation conditions.

**Table 1:** Results of *post hoc* analyses with Holm-Bonferroni corrections for *Tonal Section* in Experiments 1 and 2.

Experiment 1			
Modulation condition	Key 1 – Transition	Key 1 – Key 2	Transition – Key 2
	*t*(24)	*t*(24)	*t*(24)
No modulation	–2.37^A^	−1.08	2.51^A^
Close modulation	−2.08	−2.31	0.88
Mid modulation	−2.69*	−2.78*	0.72
Far modulation	−3.52***	−2.09*	2.43*

Experiment 2 (no modulation condition first)			
Modulation condition	Key 1 – Transition	Key 1 – Key 2	Transition – Key 2
	*t*(14)	*t*(14)	*t*(14)
No modulation	−1.34	−1.12	0.81
Close modulation	−1.00	−1.49	−0.02
Mid modulation	−1.50	−1.61	0.36
Far modulation	−3.04*	−3.76**	−0.74

Experiment 2 (modulation conditions first)			
No modulation	−0.46	−1.76	−1.29
Close modulation	−2.11	−2.90*	0.12
Mid modulation	−0.29	−2.19	−2.68^A^
Far modulation	−4.76****	−6.25****	−2.72*

^A^*p* < 0.06; *p < 0.05; **p < 0.001; ***p < 0.005; ****p < 0.001.

### 2.3 Discussion

With respect to the principal goals of this experiment, the current results present a mix of both anticipated and unexpected findings. In terms of the expected results, there is an effect of modulation on pianists’ abilities to accurately sight-read a passage of music. This impact was evident for the (averaged across modulation condition) pairwise comparisons of all three sections: Key 1 and Transition sections, Key 1 and Key tonal sections, and Transition and Key 1 sections. Considered in a global sense, these differences align with our second and first predictions (respectively) highlighted earlier, and are what might be expected from a perception-action framework for sight-reading. The necessity of shifting perceptual and motor representations produced by modulation caused a disruption in performance, producing increased error rates at the transition point between the keys, as well as increased errors between the initial and subsequent sections.

In line with our third prediction, there also appears to be an effect of the degree of movement in tonal space on performance. Specifically, when looking at the individual modulation conditions, the far modulation condition consistently produced significant differences across the various sections. In contrast, in the no modulation condition, although there appears to be an increase (albeit non-significant) in error rates at the transition section, there was no difference between the two key areas of these passages. The close and mid modulations fall between these two sets of patterns, with the close modulations producing no differences between the sections, and the mid modulations producing differences between two of the three sections. These results largely align with predictions regarding the effect of distance of tonal modulation on error rates in performance, with the most disruption in performance observed for the farthest distance, and decreasing disruption with decreasing tonal distance.

One unexpected result of this study was the increase in error rates at the transition point in the no modulation condition. Although this increase was not significant, the pattern is nevertheless striking given that, because there was no modulation in these stimuli, there is no logical reason for performance accuracy to have varied at all across these sections; all sections (Key 1, Transition, Key 2) were literally in same key. As such, there simply should not have been any change in error rates observed across these melodies. One of the principal goals of Experiment 2 was thus to investigate this perplexing result.

## 3 Experiment 2: sight-reading of modulating melodies: abstraction of anticipated tonal movement

One reason that the no modulation melodies of the previous experiment might have demonstrated unexpected increased error rates lies in the decision to randomly order the stimulus melodies in this study. Although random presentation of stimuli is generally considered a *sine qua non* of good experimental design, in the current case this procedure led to participants experiencing experimental sessions in which three-quarters of the trials had modulating melodies, and one-quarter had non-modulating melodies. One possibility is that this high prevalence of modulating passages may have biased performers to generally anticipate key movement in these stimuli. Although this expectation would be unfulfilled for the no-modulation melodies, it could have nevertheless led to increased errors at the transition position through a variety of mechanisms, including performers actively inhibiting the previous key section, misreading of the notes in scores, and so on.

Of course, this explanation assumes that participants in an experiment actively abstract structural regularities from a set of presumably independent individual trials in an experiment, with these regularities then guiding responses in the experimental context. In fact, recent work by Schmuckler et al. (2020) uncovered evidence for exactly such a process. Presented as a set of case studies, this paper highlighted how the unintended tonal structure of a set of stimuli, discernible only as an abstract property across a series of independent trials, each of which was individually devoid of this overall structure, nevertheless influenced participants’ responses in these contexts. The two experimental contexts producing these effects differed dramatically, with the first involving pianists’ performances of two-note dyads (based on work by Schmuckler and Bosman, 1997), and the second involving listeners’ expectancy generation and memory for tones in short atonal melodies (based on work by Vuvan et al., 2014). Without going into the details of these analyses (see Schmuckler et al., 2020, for a detailed review and discussion), this work demonstrated the feasibility of abstraction of overall structural properties, and specifically tonal structure, across a series of trials in both performance and perception contexts. Accordingly, the idea that performers in Experiment 1 anticipated key modulations in these stimuli seems quite possible. One of the principal goals of this study was to examine this hypothesis.

This study also provided an opportunity to test the impact of a (belatedly recognized) potential confound in the stimuli of the previous experiment. Specifically, it was realized that the stimuli employed in Experiment 1 contained a relation between the tonal distance of the modulating melodies and the tendency for the written notation of the melodies to contain a mixed set of accidentals (i.e., the use of both flats and sharps in the melodies). For the no modulation and close modulation condition there were no occurrences of mixed accidentals in the stimulus scores. However, the 12 mid-modulation melodies contained four melodies with mixed accidental scores, and the far modulation stimuli contained 10 melodies with mixed accidental scores. Although the occurrence of both flats and sharps in a musical score is a common occurrence naturalistically, their presence could have nevertheless created additional attentional demands for performers, above and beyond that needed for the perceptual-motor requirements of sight-reading. These additional demands may have thus contributed to, or even driven, the observed increase in errors across these conditions. Accordingly, it is of interest to examine the impact of this factor on the tonal distance effects just observed.

Experiment 2 addressed both of these aspects. To evaluate whether exposure to modulating sequences generates modulatory expectations in the no modulation sequences, we simply blocked the presentation order of the different conditions. Specifically, one group of participants completed all the no-modulation condition trials prior to encountering any of the other modulation conditions, whereas a second group completed the no modulation trials after experiencing the modulation conditions. Comparing performance between these two groups allows for a direct assessment of whether prior experience with modulating sequences drives expectations in non-modulating contexts. If such exposure induces an expectation of modulation, then error rates in the Transition section, and potentially the Key 2 section, should be reduced for the first group relative to the second group of performers. To address concerns regarding mixed accidentals, this issue can be eliminated by employing enharmonic note spellings (e.g., replace Bb with A#) whenever necessary to equate the types of accidentals across the two tonalities. Although this approach introduces some unconventional instances of musical notation (as discussed in the upcoming methods section), it offers a straightforward solution to this issue.

### 3.1 Methods

#### 3.1.1 Participants

Thirty trained pianists (22 females; mean age = 20.56 years, *SD* = 1.63 years) were recruited from the University of Toronto Scarborough population. Participants received either extra credit in a psychology course or a $30 Amazon Gift card for participating. As with Experiment 1, eligibility requirements included having a minimum of Grade 7 sight-reading ability using guidelines established by the Royal Conservatory of Music or an equivalent musical institution. Participants reported an average of 15.33 (*SD* = 3.32) years of playing the piano, 10.93 (*SD* = 3.82) years of formal instruction, 2.78 (*SD* = 3.02) hours of weekly practice, with a modal level of 10 for their highest certificate in piano performance.

#### 3.1.2 Materials, apparatus, experimental design, and procedure

With respect to the stimulus materials, the principal variation from the previous experiment involved modifying the to-be-performed musical scores to remove all instances of mixed accidentals (i.e., both flats and sharps) in a given musical score, when necessary. These changes were accomplished via the use of enharmonic note spellings for one of the two component tonalities in the passage. Figure 5 shows the implementation of this process of harmonizing accidentals for the previously viewed sample melodies, explicitly indicating the occurrence of enharmonic note usage.

**Figure 5:**
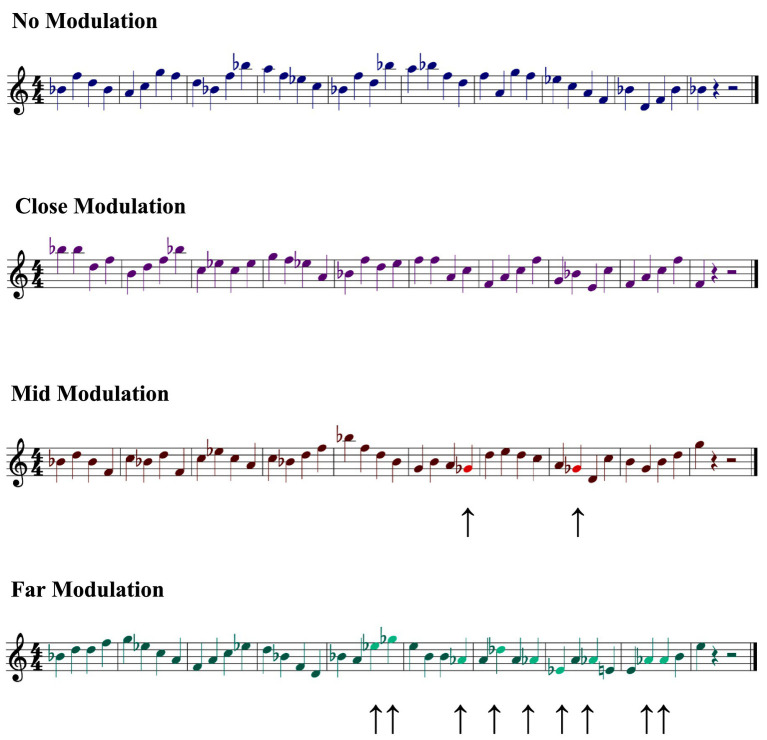
Sample melodies in the no modulation, close modulation, mid modulation, and far modulation conditions in Experiment 2. Harmonized accidentals are indicated with arrows (↑).

It should be noted that employing enharmonic notations does produce unusual note collections within a music-theoretic framework. For instance, the far modulation stimulus shown in Figures 2, 3, and 5 comprises movement from Bb major to E major. Bb major employs two flats (Bb and Eb), whereas E major contains four sharps (F#, C#, G#, D#). One way of equating the accidentals across these two sections would be to use the enharmonic key for one of the two – either A# major for Bb major, which would change the flats to sharps, or Fb major for E major, which would change the sharps to flats. Although both of these enharmonic tonalities are technically possible, neither are especially practicable, given that both are unusual and somewhat novel keys for performers. As an alternative, rather than employing the entire enharmonic tonality, it is also possible to modify only the notes containing the mixed accidentals for one of the two tonalities. In the previous example, this would mean changing either the Bb and Eb to A# and D# in the Bb major tonality, or the F#, C#, G#, and D# to Gb, Db, Ab, and Eb, in the E major tonality. In this case, although modification of Bb major requires fewer changes, because we attempted to roughly balance the proportion of melodies written with flats and sharps, we determined it was better to employ the enharmonic spellings for the E major notes. As an aside, it is recognized that this procedure does create an unusual spelling of the diatonic set (E, Gb, G, Ab, A, B, Db, Eb, E). However, because none of the melodies contained key signatures, or spelled out the diatonic set directly, the impact of this oddity was judged to be minor.

The critical design modification of this study involved using a blocked design for stimulus presentation, with two groups of participants. The first group (*N* = 15), called the “no modulation condition first” group, received a random ordering of the no modulation melodies in a single block of 12 trials, presented at the beginning of the experiment. After completing these trials, the remaining 36 trials for the close, mid, and far modulation conditions were then randomly presented. The second group (*N* = 15), called the “modulation conditions first” group, received a block of the randomly ordered 36 trials for the close, mid, and far modulation conditions initially, followed by a block of the randomly ordered 12 no modulation melodies. Note that this design fails to examine the impact of the degree of exposure to modulating melodies on performance (testing this question would require a Latin square design consisting of all counterbalanced orderings of conditions); however, because our question was focused only on whether experience with modulating melodies produced an expectation for such modulation, such a design, although more nuanced, is significantly less efficient. Accordingly, we restricted our focus to the basic issue of having or not having experienced modulation melodies. All remaining aspects of the experimental apparatus and procedure were identical to Experiment 1.

### 3.2 Results

Performances were preprocessed equivalently to Experiment 1, resulting in average error rates for all performers as a function of individual measures, ultimately aggregated into the three sections of Key 1, Transition, and Key 2, for all four modulation types. Error rates were analyzed in a 3-way ANOVA, with the within-subjects factors of *Tonal Section* (Key 1, Transition, Key 2) and *Modulation Condition* (no modulation, close modulation, mid modulation, far modulation), as well as the between-subjects factor of *Condition Order* (no modulation condition first, modulation conditions first). Initially analyses found that, based on the Shapiro–Wilk test, normality was violated for 6 of the possible 24 combinations of variables in this study. Accordingly, given the robustness of ANOVAs with respect to such violations (Schmider et al., 2010), and the fact that these normality violations occurred for only a handful of our means, we elected not to transform the data. Additionally, Mauchley’s test indicated that sphericity was violated for the main effects of *Tonal Section* (*p* = 0.003) and *Modulation Condition* (*p* = 0.004), as well as the *Tonal Section* x *Modulation Condition* interaction (*p* < 0.001). Accordingly, Greenhouse–Geisser corrections were applied to the degrees of freedom for these factors. Finally, this study observed a violation of the assumption of homogeneity of variance, based on Levene’s test for equality of variance. Fortunately, one consequence of the application of the Greenhouse–Geisser correction is that it also addresses violations of homogeneity of variance.

Figure 6 shows errors rates (and SEs) as a function of the three factors noted above. The ANOVA revealed main effects for *Tonal Section*, *F*(1.48,41.38) = 19.18, *MSE* = 69.50, *p* < 0.001, *partial η^2^* = 0.41, and *Modulation Condition*, *F*(2.14,51.80) = 41.65, *MSE* = 43.18, *p* < 0.001, *partial η^2^* = 0.60, but no main effect for *Condition Order*, *F*(1,28) = 0.86, *MSE* = 675. For the main effect of *Tonal Section*, *post hoc* tests with Holm-Bonferroni corrections revealed significant differences between the Key 1 (*M* = 10.34, *SE* = 1.08) and Transition (*M* = 14.45, *SE* = 1.50) sections, *t*(28) = −4.80, *p* < 0.001, as well as Key 1 and Key 2 (*M* = 15.84, *SE* = 1.76) sections, *t*(28) = −4.79, *p* < 0.001. The difference between the Transition and Key 2 sections was marginally significant, *t*(28) = −2.05, *p* = 0.05. For the main effect of *Modulation Condition*, errors increased systematically across the no modulation (*M* = 10.17, *SE* = 1.19), close modulation (*M* = 12.18, *SE* = 1.46), mid modulation (*M* = 12.92, *SE* = 1.43), and far modulation (*M* = 18.93, *SE* = 1.71) conditions. *Post hoc* tests with Holm-Bonferroni corrections revealed significant differences between all pairwise comparisons, with the exception of the close and mid modulation conditions. Of the remaining effects, there was a significant *Tonal Section* x *Modulation Condition* interaction, *F*(3.60,100.92) = 9.96, *MSE* = 34.52, *p* < 0.001, *partial η^2^* = 0.26.

**Figure 6:**
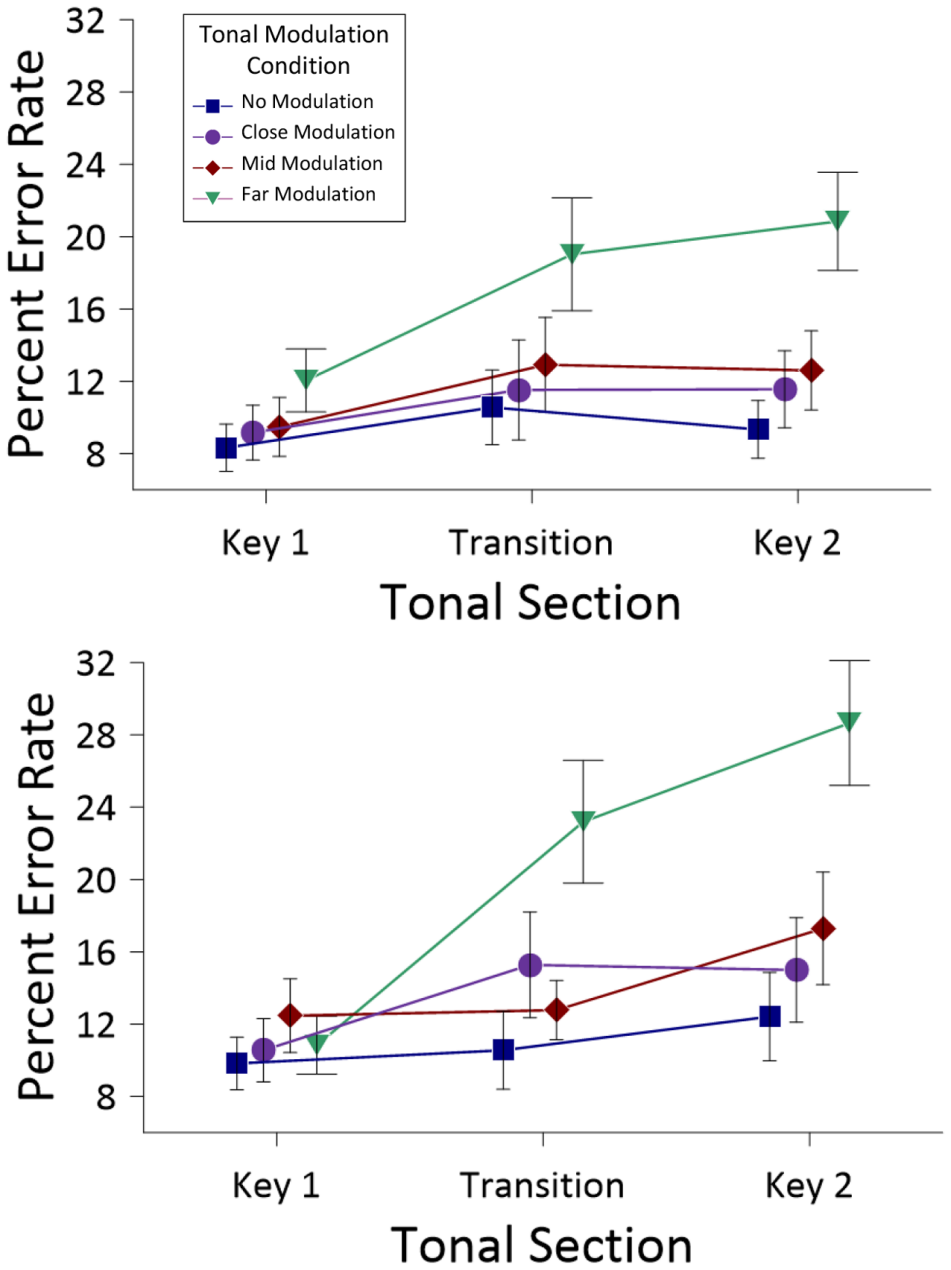
Percent error (and SEs) for the *Tonal Modulation* conditions, as a function of *Tonal Section*, in the no modulation condition first (top) and modulation conditions first (bottom) for Experiment 2. This figure is presented for information only; the three-way interaction was not significant.

Again, although the three-way interaction was non-significant, *F*(3.60,100.92) = 1.75, *MSE* = 34.52, *n.s.*, as with the previous study, a series of follow-up analyses using Holm-Bonferroni corrections examined the pattern of error rates as a function of *Tonal Section*, for the no modulation condition first and modulation conditions first groups separately, with respect to the individual modulation conditions. The results of these analyses also appear in Table 1, and demonstrate a comparable pattern as to what has already been described. For the participants in the no modulation first condition, there were no significant differences between any of the sections in the no modulation condition, nor were there any differences between the sections for the close and mid modulation conditions. For the far modulation condition, however, errors in the Key 1 section were significantly lower than error rates in the transition and the Key 2 section, with no difference in error rates between the Transition and Key 2 section.

For modulation conditions first participants, in the no modulation condition there was no difference in error rates between the Key 1, Transition, and Key 2 sections of these melodies. For the close and mid modulation conditions, differences in error rates between the sections began to appear, with the Key 1 section producing lower error rates than the Key 2 section for the close modulation condition, and a non-significant trend for the mid modulation condition. Finally, in the far modulation condition, the differences between the sections become consistent, with the Key 1 section significantly lower than both the Transition and Key 2 sections, with a significant increase in errors between the Transition and Key 2 sections as well.

#### 3.2.1 Cross-experiment analyses

A final analysis examined error rates during sight-reading across the two experiments. This analysis is of interest in that it provides a direct assessment of the impact of the two principal modifications employed in Experiment 2 – the blocking of no modulation and modulation trials, and the impact of harmonizing accidentals across the passages, on sight-reading performance. In this analysis, percent error rates were analyzed in a three-way ANOVA, with the within-subjects factors of *Tonal Section* (Key 1, Transition, Key 2), *Modulation Condition* (no modulation, close modulation, mid modulation, far modulation), and the between-subjects factor of *Stimulus Presentation* (intermixed [Experiment 1], no modulation condition first [Experiment 2], modulation conditions first [Experiment 2]). Mauchley’s test indicated that sphericity was violated for the main effects of *Tonal Section* (*p* = 0.051) and *Modulation Condition* (*p* = 0.01), as well as for the *Tonal Section* x *Modulation Condition* interaction (*p* < 0.001). Accordingly, Greenhouse–Geisser corrections were applied to the degrees of freedom for these factors.

The above ANOVA revealed main effects for *Tonal Section*, *F*(1.80, 93.67) = 27.80, *MSE* = 46.84, *p* < 0.001, *partial η^2^* = 0.35, and *Modulation Condition*, *F*(2.53,131.58) = 46.99, *MSE* = 28.55, *p* < 0.001, *partial η^2^* = 0.48, but no main effect for *Stimulus Presentation*, *F*(2,52) = 1.06, *MSE* = 859.30, *n.s*. For the main effect of *Tonal Section*, *post hoc* tests with Holm-Bonferroni corrections revealed significant differences between the Key 1 (*M* = 9.89, *SE* = 0.94) and Transition (*M* = 13.87, *SE* = 1.37) sections, *t*(52) = −5.96, *p* < 0.001, as well as the Key 1 and Key 2 sections (*M* = 14.14, *SE* = 1.33), *t*(52) = −6.01, *p* < 0.001, but not between the Transition and Key 2 sections, *t*(52) = −0.52, *n.s*. For the main effect for *Modulation Condition*, *post hoc* texts revealed a systematic increase in errors across the no modulation (*M* = 10.23, *SE* = 1.13), close modulation (*M* = 11.56, *SE* = 1.20), mid modulation (*M* = 12.27, *SE* = 1.19), and far modulation (*M* = 16.47, *SE* = 1.36) conditions, with significant differences between almost all modulation conditions (*p* < 0.001 for all comparisons), except between the no modulation and close modulation conditions, *t*(52) = −2.55, *n.s.*, and between the close and mid modulation conditions, *t*(52) = −1.46, *n.s*.

This analysis also revealed significant two-way interactions between *Tonal Section* and *Modulation Condition*, *F*(4.23,219.79) = 11.18, *MSE* = 23.65, *p* < 0.001, *partial η^2^* = 0.18, *Modulation Condition* and *Stimulus Presentation*, *F*(5.06,131.58) = 9.17, *MSE* = 28.55, *p* < 0.001, *partial η^2^* = 0.26, and *Tonal Section* and *Stimulus Presentation*, *F*(3.60,93.67) = 3.79, *MSE* = 46.85, *p* = 0.006, *partial η^2^* = 0.13. These two-way interactions were qualified by a significant three-way interaction between all factors, *F*(8.45,219.79) = 3.23, *MSE* = 23.65, *p* < 0.001, *partial η^2^* = 0.11. This interaction can be seen by comparing the pattern of findings across Figures 4 and 6. To determine the locus of this interaction, a series of *post hoc*, one-way univariate analyses compared percent error rates as a function of the three stimulus presentation modes (intermixed, no modulation condition first, modulation conditions first) for each individual combination of *Tonal Section* and *Modulation Condition* (nine analyses in total: Key 1, no modulation; Transition, no modulation … Key 2, far modulation). Of these comparisons, the only significant difference across the three stimulus presentations was for the Key 2, far modulation combination, *F*(2,52) = 9.68, *MSE* = 134.20, *p* < 0.001, *partial η^2^* = 0.27. *Post hoc* pairwise comparisons using Holm-Bonferroni corrections revealed significant differences between the error rates with the intermixed (*M* = 11.50, *SE* = 2.32) and the no modulation condition first stimulus presentations (*M* = 20.85, *SE* = 2.99), *t*(52) = −2.50, *p* = 0.032, as well as the intermixed and modulation conditions first stimulus presentations (*M* = 27.65, *SE* = 2.99), *t*(52) = −4.29, *p* < 0.001, but not between the latter two conditions, *t*(52) = −1.61, *ns*. Accordingly, the locus of this interaction arises for the second tonality in the far modulation condition, with the intermixed stimuli of Experiment 1 leading to significant fewer errors than the different forms of blocked presentations employed in Experiment 2.

### 3.3 Discussion

The current findings provide insight into the initial questions driving this study. Regarding the impact of prior exposure to modulation on performers’ expectations, this study provides evidence that such experience shapes processing during the sight-reading of modulating melodies. Specifically, for the no modulation condition there was no evidence of any change in error rates across the different sections of these melodies. This result stands in contrast to Experiment 1, in which the no modulation melodies produced a pattern of (non-significant) errors across the sections – a perplexing result given that these melodies did not change in their tonal information across their extents. As previously discussed, the most viable hypothesis for such a result is that, regardless of the actual content of the passages, performers developed expectations that modulations would occur, with these expectations then influencing performance. This result fits with previous work demonstrating that tonal information plays a significant role on performers’ expectations for, and productions of, musical passages quite generally (Schmuckler, 1989, 1990). Even more interestingly, and as a cautionary note, these results provide yet another example of how stimulus information aggregated across multiple independent trials of an experiment can significantly effect, sometimes deleteriously, the results of that experiment; this finding has been discussed by Schmuckler et al. (2020).

Although finding that initial presentation of the no-modulation melodies in a concentrated block eliminated performers’ expectations for modulation in these passages, this effect occurred when the no-modulation melodies were presented (as a block) both prior and subsequent to the modulation melodies. This result adds a nuance to the operation of expectation generation within the current context, showing that performers’ expectations can also change adaptively over the course of an experimental session. Thus, although performers in the modulation conditions first group likely began the final set of no modulation trials expecting to encounter modulating melodies, they likely modified this expectation after some exposure to these trials, now anticipating that the stimuli would remain in the same key across their length.^3^ This finding also converges with the analyses of Schmuckler et al. (2020) in showing that the abstraction of aggregate structure can occur powerfully over short periods of exposure to stimuli. In their original paper, Schmuckler et al. simulated the number of trials needed to abstract the presumed tonal structure in both of their case studies, with these simulations suggesting that such structure was available within relatively few trials (7 and 16 trials, respectively, in the two case studies). The current studies support these simulations, demonstrating structural abstraction, whether initially or adaptively, within 12 trials of exposure.

As for the second goal of this study – examining the impact of harmonizing accidentals across changing tonal sections – this study found that error rates for the modulating melodies continued to be influenced in a generally comparable fashion as Experiment 1. Thus, the impact of tonal distance observed in the previous study was not simply an artifact of visual confusion arising from the use of mixed accidentals.

This is not to say that modifying the accidentals had no effect on performance, however. In fact, this study found an even stronger influence of modulation on sight-reading. Specifically, error rates increased in the transition, and remained elevated throughout, with the Key 2 section of the far modulation melodies producing elevated error rates with respect to the initial key and the transition section. One possible explanation for this increased effect is that, rather than confusing performers, the mixed accidentals in Experiment 1 actually provided a clearer indication that the passage had modulated to a new key. Accordingly, performers more successfully adaptively modified their expectations for what notes should occur with respect to this new tonality, thereby reducing error rates. In contrast, in Experiment 2, without an obvious indication of key change arising from changed accidentals, performers were slower to adapt to this new key, elevating errors in the subsequent tonal section. Although this finding was unexpected, in retrospect its occurrence is consistent with the perception-action processes presumably underlying performance in this context.

## 4 General discussion

With respect to the principal motivations for this project, these results provide compelling evidence that the sight-reading of melodies incorporating a change of key lead to increased performance errors subsequent to the key change. Specifically, this study observed an increase in error rates at the transition section between the two keys, a finding in keeping with the first prediction articulated for these results. Additionally, this study found that error rates continued to be elevated in the Key 2 section, relative to the first key, a finding in keeping with the second prediction previously discussed. Intriguingly, one as yet unaddressed aspect of this second result involves the time scale of this continued disruption. Clearly one would not expect that modulation would have an unending impact on performance; ultimately, performers will adjust to the new key, and sight-reading errors will drop to previous baseline levels. However, the time frame for such adaptation remains unknown. This issue could be easily tested by providing differing lengths of new key information, and tracking accuracy as a function of passage length.

Finally, this study also observed that the strength of the disruptions induced by tonal modulations was itself moderated systematically by the distance, in tonal space, of these modulations. In keeping with the third prediction delineated, this study found that the greater the tonal distance between the initial and subsequent key, the more significant were the performance disruptions. This demonstration of tonal distance effects in sight-reading complements research examining tonal distance effects in listeners’ percepts of modulating passages (Cuddy and Thompson, 1992; Lewandowska, 2019; Lewandowska and Schmuckler, in preparation; Thompson and Cuddy, 1989, 1992, 1997). Generally, this perceptual work demonstrates that listeners are sensitive to the tonal distance of modulating passages, as assessed by direct ratings of perceived distance (Thompson and Cuddy, 1989, 1992, 1997) and ratings of the musical fit of, and processing times for, target chords related to or unrelated to the initial versus subsequent key sections of modulating passages (Lewandowska, 2019; Lewandowska and Schmuckler, in preparation).

On a fundamental level, all of these findings are understandable with respect to a perception-action framework of motor control in piano performance, discussed earlier (Maes et al., 2013; Novembre and Keller, 2014; Pfordresher, 2006, 2019; Schaefer, 2014). Along these lines, the visual and auditory (i.e., perceptual) information from the beginnings of these melodies generate schematic representations of the tonal structure of these passages. Given the explicit sight-reading context, these perceptual schemas produce complementary motor schemas involving specific scalar patterns to be performed, consistent with the implied tonal structure of these passages. Although speculative, given the highly overlearned and well-practiced status of tonal scales for musicians (and particularly pianists), it seems reasonable that identification of a specific tonal context would give rise to motor expectations for producing certain specific notes (e.g., diatonic scale tones). In other words, recognition of a given tonal context leads to the operation of perceptual and motor schemas consistent with this tonal context.

Modulation of the melodies to a new key, therefore, requires modifying both perception and action schemas, leading to sharp increases in performance errors at the transition point, and relatively elevated errors for the new key. The tonal distance effect arises because more distant modulating keys literally have less related perceptual, and (presumably) motor schematic representations. Although the second part of this idea (the relation between motor schemas) is a supposition, current psychological models of key relations and tonal processing (Bharucha, 1987, 1989; Bharucha and Todd, 1989; Collins et al., 2014; Tillman et al., 2000) provide ample support for the first part of this idea (less related perceptual/cognitive representations).

This framework also accounts for an unanticipated result of this study: the greater and more prolonged disruption of performance for the Key 2 section in Experiment 2, relative to Experiment 1, as indicated in the cross-experiment analyses. In this case, and as discussed earlier, the difference between the studies likely arises due to the lack of change in accidentals in Experiment 2. This failure to provide an overt indicator of a key change provides less compelling information indicating the necessity of shifting representations. It is worth noting that this idea also relates to the just discussed issue of influences on the performers’ adaptation to the new tonal framework. Along with just the simple time frame for such adaptation, specific components of the musical score, such as the nature of accidentals, could also influence performers’ adoption of these new key schemas. Although hypothetical, this idea could be tested by having performers sight-read the same modulating melodies notated with changing accidentals between the two key sections, and with harmonized accidentals. If true, this idea leads to the (non-intuitive) prediction that the former notation should result in fewer errors than the latter notation.

Another assumption inherent in the perception-action framework employed in this work is that performers were activating motor schemas based on scalar representations of the perceived tonalities. Because the stimuli employed exclusively diatonic scale members in the tonal sections, this assumption seems warranted, particularly given that (as already mentioned) diatonic scales are highly overlearned patterns for pianists. This assumption leads to the prediction that employing modulating melodies in which the tonal sections contain both diatonic and chromatic tones might reduce the impact of modulation on performance, given that the presumed scale-based motor schemas would be less strongly engaged.

The current findings also raise interesting questions with respect to the understanding of psychological processes involved in perceiving modulation. One such question involves whether or not the tonal movement asymmetries observed in perceptual studies (Cuddy and Thompson, 1992; Lewandowska, 2019; Lewandowska and Schmuckler, in preparation; Thompson and Cuddy, 1989, 1992, 1997) would be similarly present in a performance context. As discussed earlier, Thompson and Cuddy (Cuddy and Thompson, 1992; Thompson and Cuddy, 1989, 1992, 1997) consistently observed an asymmetry in perceived tonal distances as a function of the direction of movement around the circle of fifths, with counterclockwise movement (e.g., G major to C major) perceived as more tonally distant that clockwise movement (e.g., C major to G major). Similarly Lewandowska (2019) and Lewandowska and Schmuckler (in preparation), in a harmonic priming context, found increased response time to counterclockwise modulations, relative to clockwise modulations. Given these results, it would be interesting to explore whether these differences in perceived tonal distance also lead to increased performance errors when sight-reading counterclockwise versus clockwise modulating melodies.

A final issue raised in this work involves its implications for what has been considered to be two of the most fundamental issues with respect to processing tonal modulations: (1) whether or not there is an enduring impact of the initial (home) key on listeners’ percepts of tonal closure, and (2) the length of time an initial key retains an impact on listeners’ percepts of a subsequent key. Currently, extant literature suggests that there is little impact of the initial key on percepts of tonal closure in a large-scale musical context (e.g., Cook, 1987), with an initial key retaining an impact anywhere from 11 s (Woolhouse et al., 2016) to 15–20 s (Farbood, 2016). In the current study, given a metronome beat of 140 BPM, the sight-read melodies lasted just under 16 s (37 notes × 428.6 msec/note = 15.86 s). Given the consistently observed tonal distance effects, these results imply a continued impact of the initial key on performance of the subsequent key, falling within the estimates of tonal influence proposed by Farbood (2016). In this regard, it would be illuminating to more systematically vary the length of these melodies, including both initial/subsequent key sections, as well as the transition section, to see if the strength of the tonal distance effect is comparably modified by such variation.

In conclusion, this work has not only provided insight into the psychological processes underlying this somewhat bespoke (and admittedly challenging) behavior, it has also helped elucidate the general perception-action mechanisms operative during skilled performance. As always with such work, the current findings represent but a step forward in our understanding of the complexities underlying such sophisticated behavior; further insights into these processes awaits future research.

## Data Availability

The raw data supporting the conclusions of this article will be made available by the authors, without undue reservation.

## Footnotes

1 Readers with a musical background will note that our choice of tonalities do not fall evenly around the circle of fifths; even spacing would have required employing tonalities of two steps, four steps, and six steps around this circle. Instead, our choice reflects a desire to employ both the most and least related tonalities for the close and far modulation conditions. Because these tonalities are one and six steps, respectively, around this circle, the decision then became whether to employ the tonality three or four steps away as the mid modulation. Ultimately, we (admittedly arbitrarily) chose the tonality three steps removed, which we would argue represents a perfectly reasonable moderately related tonality.

2 One might ask why we chose the intermediate step of calculated percent errors on a measure-by-measure basis and then aggregate these errors into sections, as opposed to simply calculating percent errors on the aggregated sections directly. Our initial intent was to analyze the data on a measure-by-measure basis; unfortunately, this analysis did not provide any additional insight into response patterns beyond what was discernible from the sectional analyses. Accordingly, in the interests of efficiency, clarity, and brevity, we present only the sectional analyses.

3 In fact, we attempted to quantify this adaptation process by examining percent error rates in this block on a trial by trial basis. Unfortunately, this microanalysis led to inconclusive findings.
